# Comparison of the Pediatric Sequential Organ Failure Assessment (p SOFA) Score and Lactate Clearance as Predictors of Morbidity and Mortality in Pediatric Sepsis: A Prospective Observational Study

**DOI:** 10.7759/cureus.79172

**Published:** 2025-02-17

**Authors:** Bharti Aggarwal, Jyoti Ranjan Behera, Amit Ranjan Rup, Reshmi Mishra

**Affiliations:** 1 Pediatrics, Kalinga Institute of Medical Sciences, Bhubaneswar, IND

**Keywords:** lactate clearance, mortality, pediatric sepsis, picu, p sofa, septic shock

## Abstract

Background: Sepsis continues to be a leading cause of illness and mortality in children around the world. Various scoring systems have been devised to predict the outcome of pediatric sepsis. Pediatric sequential organ failure assessment (p SOFA) and lactate clearance are the two commonly used methods.

Objective: The aim of this study was to compare the p SOFA score with lactate clearance as predictors of morbidity and mortality in pediatric sepsis, to compare the initial plasma lactate level and lactate clearance, and to know which is better to predict outcomes in sepsis and septic shock.

Methods: This prospective observational study was conducted in a pediatric intensive care unit of a tertiary care teaching hospital from July 2022 to June 2024. The blood lactate level and p SOFA score were assessed at admission and at 24 and 48 hours, and lactate clearance was calculated at 24 and 48 hours of admission. The receiver operating characteristic (ROC) curve was plotted to predict deaths using p SOFA, lactate level, and lactate clearance.

Results: A total of 71 children were enrolled in the study. All children were divided into two groups, 58 (82%) survivors and 13 (18%) non-survivors. The most common diagnosis was pneumonia, observed in 31 (43.6%) children. Compared to survivors, non-survivors had a higher prevalence of multiple organ dysfunction syndrome (MODS). The most common organ system involved was the cardiovascular, in 50 (70%) cases. For predicting mortality, p SOFA scores were statistically significant at admission and at 24 and 48 hours with a high area under the curve (AUC) at 48 hours (0.985). Lactate clearance at 24 hours was a better predictor of mortality than at 48 hours with a higher AUC (0.958).

Conclusion: Both p SOFA score at 48 hours and lactate clearance at 24 hours were significant predictors of mortality. Among both parameters, lactate clearance at 24 hours was superior in predicting mortality early.

## Introduction

In children, sepsis continues to be a leading cause of morbidity and mortality worldwide [1,2]. Research reveals that mortality attributed to sepsis in pediatric intensive care units (PICUs) can exceed 50% in developing countries [3]. According to the World Health Organization, 80% of mortality of children under four years of age are sepsis-related [4]. Globally, approximately 25 million children are affected by sepsis every year, resulting in nearly three million deaths [5]. Early recognition of inadequate tissue perfusion and prompt, aggressive management are crucial in treating septic shock, especially given the rising incidence and burden of sepsis-related morbidity and mortality. Recently, lactate has been used as a key marker for assessing sepsis and patients with septic shock. Impaired lactate clearance may indicate dysfunction of renal and hepatic metabolism, which are essential for normal lactate elimination. Consequently, lactate clearance provides important insights into the host's metabolic balance and the effectiveness of resuscitation efforts [6]. The Surviving Sepsis Campaign emphasizes the reduction of serum lactate as a resuscitation target in cases having severe sepsis and septic shock [7]. Researches have demonstrated that both initial lactate levels and the rate of clearance of lactate are predictive of death in septic shock patients, with prolonged hyperlactatemia being associated with worse outcomes [8,9]. Thus, detecting raised lactate levels early can help to identify patients at higher risk of worse outcomes and enable timely interventions. Assessing prognosis and illness severity at admission is crucial. Hence, the best prognostic scores should be accurate, easy to understand, affordable, and minimally invasive. In this research, we used the pediatric sequential organ failure assessment (p SOFA) scoring system [10]. The aim of our study was to compare the p SOFA score with lactate clearance as predictors of morbidity and mortality in pediatric sepsis, to compare the initial plasma lactate level and lactate clearance, and to know which one is better to predict outcomes in sepsis and septic shock.

## Materials and methods

This prospective observational study was conducted in the PICU of the Department of Pediatrics, Kalinga Institute of Medical Sciences, Bhubaneswar, Odisha, India, over a period of two years (July 2022 to June 2024). The Institutional Ethics Committees reviewed and approved the current study protocol. Before each patient was enrolled, their parents or guardians gave their informed written consent. The patients enrolled were children aged between one month and 18 years having sepsis and septic shock, admitted to the PICU. The definitions of sepsis and septic shock were according to the International Pediatric Sepsis Consensus Conference 3 (IPSCC) criteria [11]. Children having malignancies, chronic medical illnesses (chronic liver disease or chronic kidney disease), or an inborn error of metabolism or those on any immunosuppressive medication were excluded. Cardiogenic, hemorrhagic, and obstructive shock patients were excluded. Post-surgical cases, poisoning cases, and patients having PICU stay for less than 24 hours were also excluded.

Detailed socio-demographic data, history, physical examination, and systemic examination were recorded within one hour of admission. Investigations like complete blood count (CBC), procalcitonin, c-reactive protein (CRP), renal function tests, liver function tests, blood culture, urine culture, and arterial blood gas (ABG) analysis for lactate levels were performed at the time of hospitalization. The p SOFA score was determined within one hour of admission, and treatment was initiated as per the septic shock management protocol [10]. Vasoactive agents (epinephrine or norepinephrine) were started in case of fluid-refractory shock. All patients were treated with broad-spectrum antibiotics along with other supportive measures. ABG, lactate level (in mmol/L), and p SOFA score were reassessed at 24 and 48 hours. Lactate clearance was calculated by using the following formula:

\[ \text{Lactate Clearance} = \left( \frac{\text{Lactate}_{\text{admission}} - \text{Lactate}_{\text{24h or 48h}}}{\text{Lactate}_{\text{admission}}} \right) \times 100 \]

Every patient was followed up until they were discharged from the PICU or died in the hospital. Following Shapiro-Wilk test's assessment of the normality assumption, all continuous variables were reported as the mean ± standard deviation (SD) or median (Q1-Q3), and categorical variables as frequency and percentage. The Student t-test and Mann-Whitney U-test were used to compare survivors and non-survivors. The chi-squared test was used to measure the relationship between category variables. The receiver operating characteristic (ROC) curve was plotted to predict deaths using p SOFA, lactate level, and lactate clearance. IBM SPSS version 23.0 (IBM Corp., Armonk, NY, US) was used for the statistical analysis, and a p-value of less than 0.05 was considered statistically significant.

## Results

A total of 71 children were included in the study. Of these participants, there were 44 (62%) male children and 27 (38%) female children, with a male-to-female ratio of 1.6:1. The mean age of the study population was 9.7 (±5.05) years. For analysis, all children were divided into two groups, survivors 58 (82%) and non-survivors 13 (18%). Among the survivors, there were 37 male children and 21 female children, while among the non-survivors, there were seven male children and six female children. The highest mortality was seen among the one year to five years of age group, which was 40%. Age or gender was not statistically significant in predicting mortality.

The most frequently observed clinical presentation included fever, followed by cough, hurried breathing, loose stool, vomiting, and seizure. The most common diagnosis was pneumonia, which was found in 31 (43.6%) children. Culture-positive sepsis was identified in 16 (22%) cases. The most frequently isolated organisms were *Acinetobacter baumannii* in five (31%), *Staphylococcus aureus* in three (19%), *Salmonella paratyphi* in two (13%), and *Escherichia coli* in two (13%), with *Klebsiella pneumoniae*, *Streptococcus pneumoniae*, *Pseudomonas*, and *Moraxella* each in one (6%) case. Out of these 16 cases, 12 survived and four died. According to our study, no significant association was observed between culture-positive sepsis and mortality. A comparison of other demographic and laboratory parameters between survivors and non-survivors is summarized in Table 1.

**Table 1:** Organ dysfunction in survivors and non-survivors Expressed in percentage (%), chi-squared test statistic (χ^2^) ^*^Expressed in median (Q1-Q3), Mann-Whitney U-test Hb: hemoglobin; GCS: Glasgow coma scale; TPC: total platelet count; ULN: upper limit of normal; mg/dL: milligram per deciliter; gm/dL: grams per deciliter; μL: microliter

Organ system involvement	Total n = 71 (%)	Survivors n = 58 (%)	Non-survivors n = 13 (%)	χ^2^/U-value	p-value
Severe anemia (Hb < 7 gm/dL)	8 (11.2%)	6 (10.3%)	2 (15.4%)	0.270	0.603
Leukopenia	11 (15.5%)	8 (13.8%)	3 (23.1%)	3.313	0.208
Leukocytosis	32 (45%)	29 (50.0%)	3 (23.1%)	3.313	0.208
Thrombocytopenia (TPC < 1.5 lakh/μL)	21 (29.5%)	18 (31.0%)	3 (23.1%)	0.323	0.569
Hepatic dysfunction (serum bilirubin ≥ 4 mg/dL)	4 (5.6%)	3 (5.2%)	1 (7.7%)	0.127	0.722
Renal dysfunction (serum creatinine > 2 times ULN for age)	11 (15.5%)	6 (10.3%)	5 (38.4%)	6.412	0.011
Neurological dysfunction GCS (≤11)	12 (16%)	5 (8%)	7 (53%)	15.465	<0.000
Numbers of patients requiring inotrope support^*^	50 (70%)	1 (0.0-2.0)	2 (2.0-3.0)	590.000	<0.000
Duration of inotrope support (days)^*^	50 (70%)	2 (0.0-4.0)	5 (2.0-7.0)	529.000	0.039
Number of days of invasive ventilation^*^	22 (30.9%)	5 (3.5-7.5)	6 (3.0-8.0)	678.000	0.969

Among all sepsis cases, 50 (70%) developed septic shock and 26 (37%) progressed to multiple organ dysfunction syndrome (MODS). Compared to survivors, non-survivors had a higher prevalence of MODS. The most common organ system involved was cardiovascular in 50 (70%) cases, followed by respiratory dysfunction in 22 (31%), hematological in 21 (30%), neurological in 12 (17%), renal in 11 (15%), and hepatocellular dysfunction in four (6%) cases. Severe anemia, thrombocytopenia, leukopenia, leukocytosis, hyperbilirubinemia, CRP, and procalcitonin levels at admission were not statistically significant in predicting mortality in cases of severe sepsis or septic shock. However, renal dysfunction was identified as a significant predictor of mortality (p < 0.05). Out of all the cases, 50 (70%) required inotrope support with 39 survivors and 11 non-survivors. Among these cases, the number of inotropes administered and the duration of inotropic support were statistically significant predictors of mortality (Table 1). Survivors and non-survivors did not significantly differ in their need for mechanical ventilation (p 0.969). Furthermore, among survivors and non-survivors, the duration of PICU stay (measured in days) was a statistically significant determinant (p 0.045) as illustrated in Table 2.

**Table 2:** Demographic and laboratory parameters ^#^Expressed in median (Q1-Q3), Mann-Whitney U-test ^*^Expressed in percentage (%), chi-squared test statistic (χ^2^) mg/dL: milligram per deciliter; gm/dL: gram per deciliter; ng/mL: nanogram per milliliter; μL: microliter; PICU: pediatric intensive care unit

Parameters	Survivors (n = 58)	Non-survivors (n = 13)	χ^2^/U-value	p-value
Age (years)^#^	12 (7-14)	9 (2-11)	256.000	0.071
Male sex^*^	37 (60%)	7 (50%)	0.4459	0.504
Hemoglobin (gm/dL)^#^	10 (8.9-10.9)	10.0 (8.8-11)	389.500	0.852
Total leukocyte count (×10^3^/μL)^#^	11.5 (6.05-19.75)	7.9 (4.5-8.6)	244.500	0.049
Total platelet count (lakh/μL)^#^	1.79 (1.37-2.89)	1.55 (1.50-2.64)	353.000	0.718
Serum bilirubin (mg/dL)^#^	0.70 (0.5-1.10)	0.60 (0.38-1.0)	336.500	0.546
Albumin (gm/dL)^#^	3.0 (2.70-3.30)	2.7 (2.3-2.9)	192.000	0.006
Urea (mg/dL)^#^	30.5 (19-45)	48 (30-62)	485.500	0.106
Creatinine (mg/dL)^#^	0.6 (0.40-0.9)	0.9 (0.7-1.10)	484.500	0.110
Procalcitonin (ng/mL)^#^	36.5 (7.9-81)	30 (9.49-74)	342.000	0.925
C-reactive protein (mg/dL)^#^	139 (57-244)	126 (64-200)	376.000	0.988
Culture positive^*^	12 (20%)	4 (30%)	0.618	0.432
PICU stay (days)^#^	3 (2-5)	6 (4-11)	510.000	0.045

The p SOFA scores were higher in non-survivors compared to survivors at admission and at 24 and 48 hours. However, the p SOFA scores remained persistently high among non-survivors over these consecutive days, with no significant changes observed. On the other hand, lactate levels were significantly higher in non-survivors compared to survivors at 24 and 48 hours, but not at admission (Table 3).

**Table 3:** Comparison of p SOFA, lactate, and lactate clearance between survivors and non-survivors ^*^Expressed in median (Q1-Q3), Mann-Whitney U-test ^#^Expressed in mean ± SD, independent t-test SD: standard deviation; p SOFA: pediatric sequential organ failure assessment; 0 hours: at admission

Parameters	Survivors (n = 58)	Non-survivors (n = 13)	t/U-value	p-value
p SOFA (0 hours)^#^	4.72 (±2.27)	10.00 (±3.06)	-7.091	<0.001
p SOFA (24 hours)^#^	3.86 (±2.36)	10.31 (±2.75)	-8.642	<0.001
p SOFA (48 hours)^#^	2.90 (±2.28)	10.17 (±2.76)	-9.684	<0.001
Lactate (0 hours)^#^	3.42 (±2.03)	3.92 (±1.56)	-0.813	0.410
Lactate (24 hours)^#^	1.82 (±1.09)	3.90 (±1.78)	-5.488	<0.001
Lactate (48 hours)^#^	1.08 (±0.66)	2.67 (±1.25)	6.183	<0.001
Lactate clearance (0-24 hours)^*^	0.45 (0.32-0.55)	0.11 (-0.35-0.17)	32.000	<0.001
Lactate clearance (0-48 hours)^*^	0.67 (0.59-0.76)	0.25 (-0.12-0.83)	215.500	0.018

**Figure 1:**
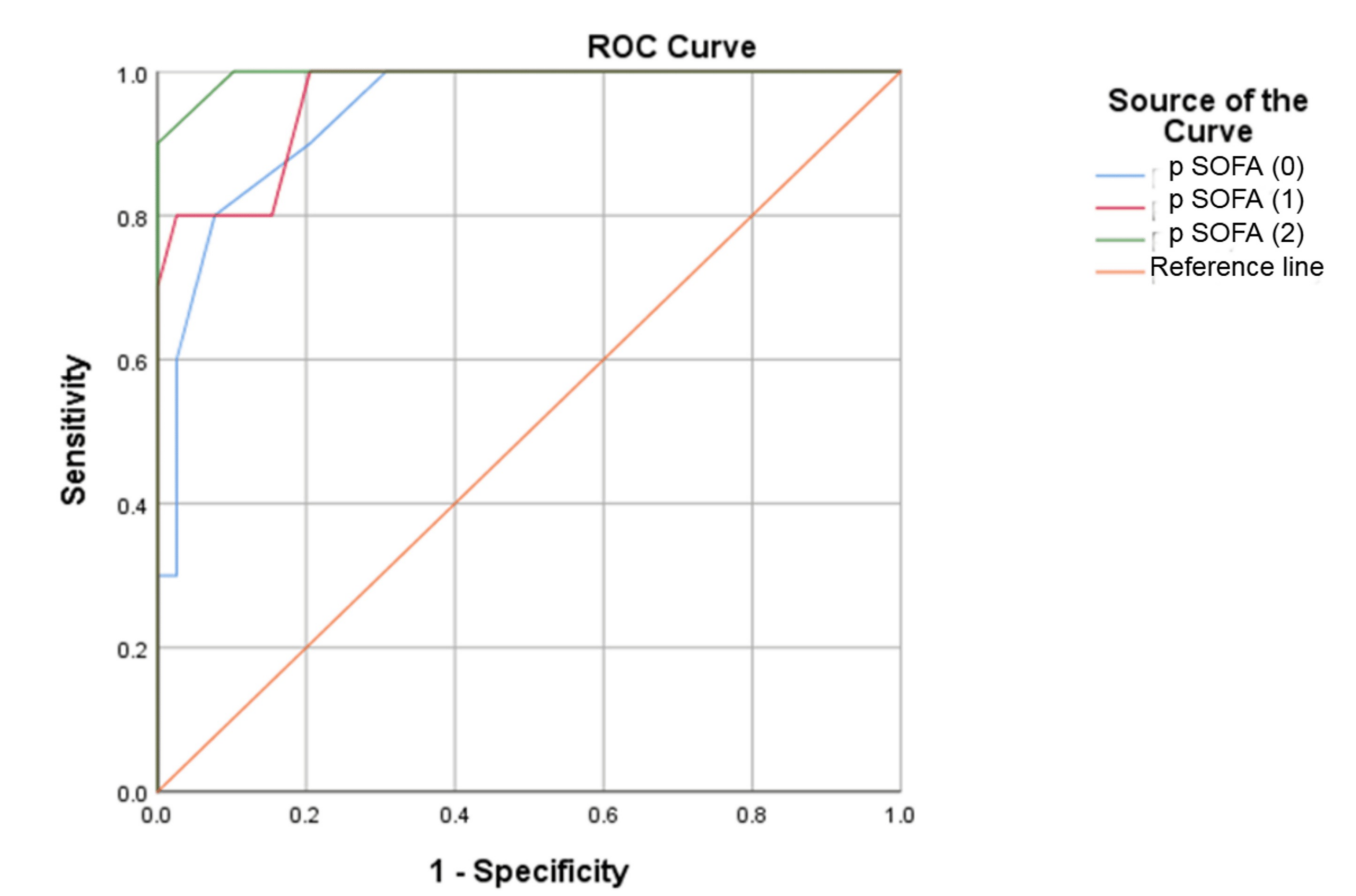
ROC curve of p SOFA at admission and at 24 and 48 hours ROC: receiver operating characteristic; p SOFA (0): ROC curve of pediatric sequential organ failure assessment at admission; p SOFA (1): ROC curve of pediatric sequential organ failure assessment at 24 hours of admission; p SOFA (2): ROC curve of pediatric sequential organ failure assessment at 48 hours of admission

**Figure 2:**
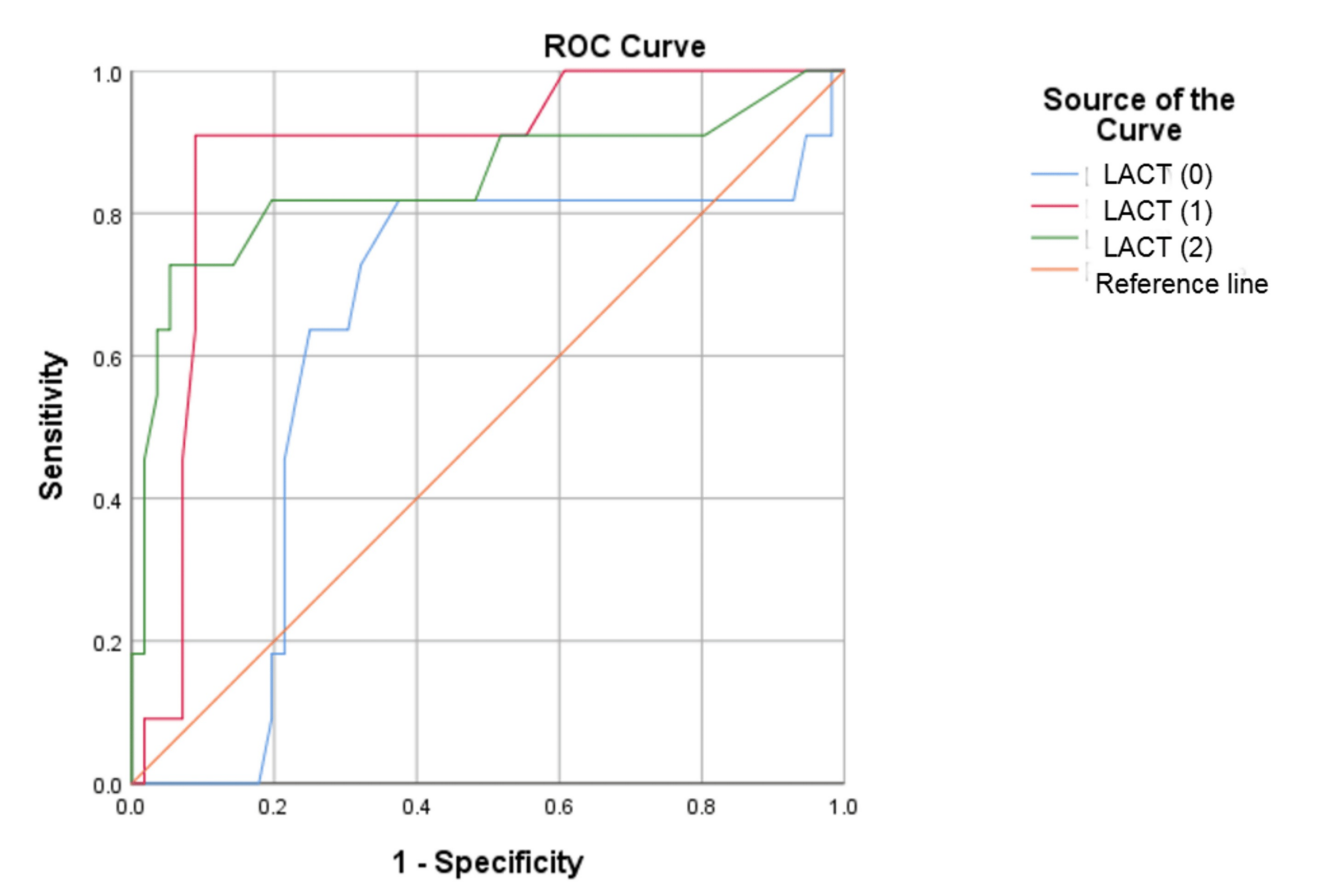
ROC curve of lactate at admission and at 24 and 48 hours ROC: receiver operating characteristic; LACT (0): ROC curve of lactate at admission; LACT (1): ROC curve of lactate at 24 hours of admission; LACT (2): ROC curve of lactate at 48 hours of admission

**Figure 3:**
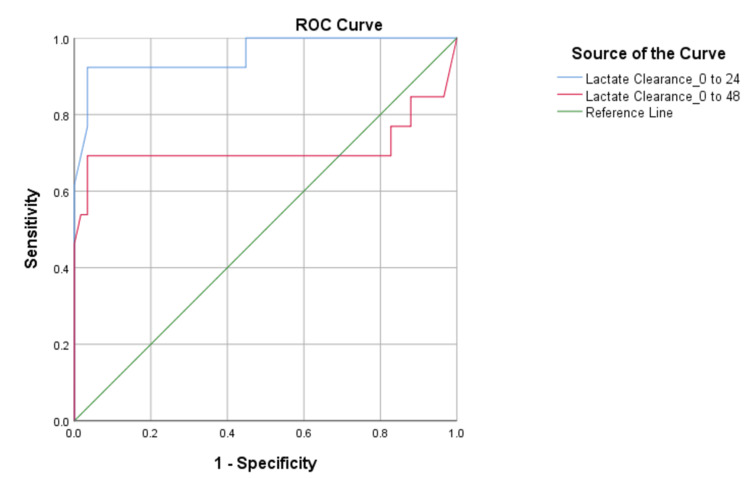
ROC curve of lactate clearance ROC: receiver operating characteristic; Lactate Clearance_0 to 24: ROC curve of lactate clearance from admission to 24 hours; Lactate Clearance_0 to 48: ROC curve of lactate clearance from admission to 48 hours

For predicting mortality, the p SOFA scores on admission and at 24 and 48 hours showed statistically significant results. During the ROC curve analysis for assessing the predictive capability of p SOFA for mortality in sepsis, the derived cutoff was 6.5 on all consecutive days (Table 4). The area under the curve (AUC) on the admission day and at 24 and 48 hours was 0.935 (95% confidence interval (CI): 0.862-1.00), 0.949 (95% CI: 0.885-1.00), and 0.985 (95% CI: 0.958-1.00), respectively. The corresponding sensitivity and specificity values were depicted (Table 4 and Figure 1). For predicting mortality, lactate levels at 24 and 48 hours of admission were found to be statistically significant (p < 0.001). However, plasma lactate measured at admission was not significantly associated with the prediction of mortality. The optimal cutoff for plasma lactate measured at 24 hours post-admission to predict mortality was 3.1 mmol/L (sensitivity of 90.9% and specificity of 91.1%) and at 48 hours was 2.15 mmol/L (sensitivity of 72.7% and specificity of 94.6%) as shown in Table 4 and Figure 2. Lactate clearance at 24 hours was significant in predicting mortality with a cutoff value of 19.5%, demonstrating a sensitivity of 92.3%, specificity of 96.6%, and an AUC of 0.958 (95% CI: 0.891-1.00, p = 0.00) represented in Figure 3. In comparison, lactate clearance at 48 hours of admission was also significant but had a lower AUC of 0.712, with a sensitivity of 69.2% and specificity of 96.6% (Table 4 and Figure 3).

**Table 4:** Comparison of the p SOFA score, lactate level, and lactate clearance to predict mortality in sepsis AUC: area under the curve; p SOFA: pediatric sequential organ failure assessment; 0 hours: at admission

Test result variable(s)	AUC	p-value	Asymptotic 95% confidence interval		Sensitivity	Specificity	Cutoff
			Lower bound	Upper bound			
p SOFA (0 hours)	0.935	0.001	0.862	1.000	100	76.800	6.500
p SOFA (24 hours)	0.949	0.000	0.885	1.000	100	83.900	6.500
p SOFA (48 hours)	0.985	0.000	0.958	1.000	100	91.100	6.500
Lactate (0 hours)	0.63	0.18	0.44	0.82	81.80	62.50	3.15
Lactate (24 hours)	0.88	0.00	0.77	0.99	90.9	91.10	3.10
Lactate (48 hours)	0.84	0.00	0.68	1.00	72.7	94.60	2.15
Lactate clearance (0-24 hours)	0.958	0.000	0.891	1.000	92.3	96.600	0.195
Lactate clearance (0-48 hours)	0.712	0.018	0.481	0.942	69.2	96.600	0.372

## Discussion

In this study, we used lactate, lactate clearance, and the p SOFA score to predict morbidity and mortality in patients with severe sepsis and septic shock. Among these parameters, the p SOFA score at 48 hours and lactate clearance at 24 hours were found to be statistically significant. Various sepsis markers such as CRP, procalcitonin, serum albumin, plasma lactate, and several mortality prognostic scores like the p SOFA score, pediatric risk of mortality III score (PRISM III), pediatric index of mortality 3 (PIM 3) score, and pediatric logistic organ dysfunction (PELOD) score are used to quantify the severity of the illness as well as estimate the probability of death [10,12-14]. The effectiveness of these predictors has been assessed in a number of researches [15,16].

We observed that female mortality rates were higher as compared to male rates, but it was statistically insignificant. Similarly, studies in pediatric populations by Kim et al. and Jaiswal et al. revealed no statistically significant association between gender and mortality [17,18]. In our study, there was no significant association between age and mortality, which is in accordance with earlier results [10,18]. However, one study reported contrasting findings with the highest mortality rate in children older than 15 years and a higher survival rate in the younger than one year age group [19]. As per our study, all age groups of children were vulnerable.

Lalitha et al. and Patil et al. identified pneumonia as the most common cause of sepsis, which was consistent with our study [20,21]. One of the earlier studies revealed higher vulnerability to MODS and increased mortality in culture-positive sepsis patients [19]. However, we observed less mortality in culture-positive children, which may be attributed to the timely administration of culture-sensitive antibiotics.

Our study indicated that the requirement of two or more inotropes for a duration of five or more days was statistically significant in predicting mortality, similar to Jaiswal et al. [18]. Other studies revealed a significant association between mechanical ventilation duration and mortality, which was not reflected in our study [18,20]. This may be attributed to fewer ventilator-associated complications and better PICU care. Renal dysfunction was a significant predictor of mortality, similar to past studies [18,20]. This indicated that children who developed acute kidney injury (AKI) faced a higher risk of morbidity and mortality. In contrast, other studies found severe anemia, thrombocytopenia, and liver dysfunction to be statistically significant predictors of mortality, which were not observed in the current study [18]. The duration of PICU stay was longer for non-survivors compared to survivors, consistent with an earlier study [22].

The p SOFA score has proven to be excellent at predicting mortality and morbidity in children with severe sepsis and septic shock, compared with other commonly used scoring systems [20,23]. The study indicated that p SOFA values at admission and 24 and 48 hours after admission were good in predicting mortality in the PICU. Non-survivors had significantly higher p SOFA scores than survivors on all three consecutive days since admission. Our results showed that 6.5 points was the ideal p SOFA cutoff for differentiating survivors from non-survivors. Few past studies have reported a higher cutoff of p SOFA for predicting mortality [10,23,24].

Our study revealed lactate levels of non-survivors were significantly greater than that of survivors at 24 and 48 hours, but not at admission. Lactate levels measured at 24 and 48 hours were more reliable predictors of mortality compared to those measured at admission. Patil et al. and Gorgis et al. similarly found no significant correlation between lactate at admission and mortality [21,25]. Jaiswal et al. observed that lactate levels in all three consecutive days were significant predictors of mortality [18]. Our results indicate that decreasing lactate levels over time are associated with improved outcomes, whereas increasing levels correlated with higher mortality risk.

Our study revealed that lactate clearance at 24 hours was more significant in predicting mortality than at 48 hours of admission. We observed higher sensitivity and specificity for almost similar cutoff values at 24 hours post-admission with much higher AUC, when compared to Jaiswal et al. [18]. Aramburo et al. and Scott et al. similarly emphasized the prognostic significance of early lactate clearance in predicting morbidity and mortality [26,27]. Patil et al. explored lactate clearance at six and 24 hours post-admission, highlighting that at 24 hours, lactate clearance achieved higher sensitivity and specificity [21]. In a meta-analysis by Zhang and Xu, lactate clearance at 24 hours was found to be a significant predictor of mortality in sepsis, which was very close to our study [28]. Our finding emphasizes the strong predictive value of lactate clearance in sepsis-related mortality, which means the higher the lactate clearance, the lower the chance of mortality.

The study identified the p SOFA score at 48 hours and lactate clearance at 24 hours as the most effective in predicting mortality in patients of severe sepsis and septic shock, both showing strong AUC values. Although the p SOFA score had a slightly higher AUC than lactate clearance at 24 hours, it is more complex and time-consuming to calculate. Considering that both the p SOFA score at 48 hours and lactate clearance at 24 hours were significant predictors, early prediction of mortality can be more practically achieved using lactate clearance at 24 hours.

Despite these excellent results, certain limitations were present in our study. With a small sample size and a constrained time frame, this study was carried out at a single center. Further studies are needed to make the study findings applicable to a larger population. Hence, multicentric trials with a larger number of cases and regular serial measurements of variables should be conducted.

## Conclusions

Our study highlights the importance of early and accurate predictors of mortality in children with severe sepsis and septic shock. The p SOFA score and lactate clearance have proven to be valuable tools in this regard. Lactate clearance at 24 hours was found to be the most effective parameter in predicting mortality in sepsis patients. A lower lactate clearance was significantly associated with worse outcomes, emphasizing its role as a dynamic marker of disease severity. Lactate clearance can help in the timely diagnosis and management of sepsis and septic shock, which will ultimately reduce the mortality rate and improve patient outcomes. Future research and multicenter studies are needed to further validate its predictive value and refine its application in clinical practice.
